# A weekly alternating diet between caloric restriction and medium fat protects the liver from fatty liver development in middle-aged C57BL/6J mice

**DOI:** 10.1002/mnfr.201400621

**Published:** 2015-01-21

**Authors:** Fenni Rusli, Mark V Boekschoten, Arantza Aguirre Zubia, Carolien Lute, Michael Müller, Wilma T Steegenga

**Affiliations:** 1 Nutrition, Metabolism & Genomics Group, Division of Human Nutrition, Wageningen UniversityWageningen, The Netherlands; 2 Norwich Medical School, University of East AngliaNorwich, UK

**Keywords:** Dietary intervention, Gene expression profile, Metabolic markers, Nonalcoholic fatty liver disease, Steatosis

## Abstract

**Scope:**

We investigated whether a novel dietary intervention consisting of an every-other-week calorie-restricted diet could prevent nonalcoholic fatty liver disease (NAFLD) development induced by a medium-fat (MF) diet.

**Methods and results:**

Nine-week-old male C57BL/6J mice received either a (i) control (C), (ii) 30E% calorie restricted (CR), (iii) MF (25E% fat), or (iv) intermittent (INT) diet, a diet alternating weekly between 40E% CR and an ad libitum MF diet until sacrifice at the age of 12 months. The metabolic, morphological, and molecular features of NAFLD were examined. The INT diet resulted in healthy metabolic and morphological features as displayed by the continuous CR diet: glucose tolerant, low hepatic triglyceride content, low plasma alanine aminotransferase. In contrast, the C- and MF-exposed mice with high body weight developed signs of NAFLD. However, the gene expression profiles of INT-exposed mice differed to those of CR-exposed mice and showed to be more similar with those of C- and MF-exposed mice with a comparable body weight.

**Conclusions:**

Our study reveals that the INT diet maintains metabolic health and reverses the adverse effects of the MF diet, thus effectively prevents the development of NAFLD in 12-month-old male C57BL/6J mice.

## 1. Introduction

The modern Western-style diet and sedentary lifestyle often promote a positive energy balance, which has importantly contributed to the global rapid increase in the prevalence of metabolic syndrome in the recent decades [1–4]. Nonalcoholic fatty liver disease (NAFLD), a condition in which the liver excessively accumulates fat, has been considered as a hepatic manifestation of metabolic syndrome. Therefore, the increasing prevalence of metabolic syndrome and NAFLD becomes a major concern [5,6] and implies an urgent need for a feasible and effective dietary intervention to prevent NAFLD [7].

Calorie restriction diet (CR) is widely known for its beneficial effects on health that is consistently demonstrated in various species [8–10], and these beneficial effects also include a decrease in hepatic triglycerides (TGs) levels [11,12]. However, an issue that has been frequently raised regarding CR is the adherence of the general population to such a strict eating pattern [13,14], as many individuals might encounter difficulties on long-term adaptation to CR after many years of habituation to a Western-style diet, as was shown by Racette et al. [15]. Therefore, a dietary regimen with alternating applications of food restriction or even food abstinence and ad libitum consumption emerges as an attractive option. Various forms of alternating dietary intervention have been explored, such as alternate-day fasting, intermittent fasting, intermittent starvation, and every-other-day feeding. The results are promising, the health benefits of the different alternative regimens are similar to continuous exposure to CR, including improved glucose tolerance and decreased cardiovascular disease risk [14,16–19]. Consequently, the application of an alternating dietary regimen as a preventive measurement against the development of NAFLD becomes of interest.

In this study, we aimed to investigate whether an every-other-week restricted diet, which we termed as the intermittent (INT) diet, is able to reverse the detrimental effects of a Western-style diet on the liver and its implication on NAFLD development by the male C57BL/6J mice. In the INT diet we applied a medium-fat (MF; 25E% fat) ad libitum diet to represent the Western-style diet and a 40E% CR diet as the restricted diet, and the animal feeding was alternated in a weekly basis between the MF and 40E% CR. To demonstrate a progressive NAFLD development by the Western-style diet, we included an intervention group exposed to continuous MF diet ad libitum. In addition, we also included two other diet groups, an ad libitum feeding of a control diet (C) and a continuous 30E% CR to complement the comparison to a normal and healthy diet, respectively. At the age of 12 months, which is representative for the middle-age time point, we examined the NAFLD progression by analyzing the metabolic, morphological, and molecular features of NAFLD.

## 2. Materials and methods

### 2.1. Ethics statement

Experiments were approved by the Local Committee for Care and Use of Laboratory Animals at Wageningen University (code number: drs-2010151b).

### 2.2. Animals and diets

Male C57BL/6J mice (age of 7 wk) were purchased from Janvier (Cedex, France) and were housed in pairs of two in the light and temperature (20°C)-controlled animal facility of Wageningen University (12 h light/dark cycle, light on at 04.00). The mice received standard AIN-93G diet [20] (Research Diet Services, Wijk bij Duurstede, The Netherlands) for 2 wk upon arrival.

At the start of the diet intervention the mice were 9–wk-old, housed individually and randomly distributed into four intervention groups: (i) control diet (C) receiving AIN-93W diet ad libitum (*n* = 89); (ii) CR receiving AIN-93W-CR in portions containing 70E% of the mean energy intake (30E% reduced energy intake) of the group of the control mice were provided each day at 15.30 (*n* = 117); (iii) MF diet (25E% fat) receiving AIN-93W-MF ad libitum (*n* = 127); and (iv) INT receiving alternating 1 wk AIN-93W-MF ad libitum followed by 1 wk 60E% based on the mean energy intake (40E% reduced energy intake) of the mice on the AIN-93W diet (*n* = 155). AIN-93W is a variant of AIN-93M (maintenance of adult mice), which slightly differs on the fat source. The 10E% fat content in AIN-93M solely comes from soybean oil, while the fat source of AIN-93W is a mix of 6E% soybean oil and 4E% palm oil, in order to balance saturated and unsaturated fat composition. AIN-93W-MF, which was used in the MF diet regimen, also contained soybean and palm oil in the same proportion. AIN-93W-CR contained increased concentration of vitamins and minerals content in order to feed these mice the same concentrations of micronutrients as the mice receiving AIN-93W diet and avoid malnutrition. Complete diet composition is listed in Supporting Information Table 1 (Research Diet Services, Wijk bij Duurstede, The Netherlands). All mice were provided with ad libitum access to water.

Body weight of all mice was recorded every 2 wk. To represent a weekly body weight development, we weighed a smaller sample of mice of each intervention group every other week (20–24 mice). Food intake of 20 mice of each intervention group was measured every 3 months, comprising 1 wk measurement for the C, CR, and MF-fed mice and 2 wk measurement for the INT-fed mice. Portion sizes of the mice on the CR and INT were adjusted at the beginning of the study and at the age of 6 months based on food intake of C mice.

At the age of 12 months, 14 mice of each intervention group were sacrificed between 14.00 and 17.00 on five consecutive days (the remaining mice stayed in the experiment to allow an investigation at older ages). To analyze an adaptive capacity of peroxisome proliferator-activated receptor alpha (PPARα), seven mice in each intervention group were treated with a PPARα agonist, while the rest of the animals were mock-treated. Prior to sacrifice each mouse was first fasted for 4 h after which they received an intragastric gavage of either solvent (0.5% carboxymethyl cellulose) or PPARα agonist Wy-14643 dispersed in solvent (160 mg Wy-14643/kg body weight), then fasted again for another 6 h. All 14 mice of each diet group were included in metabolic parameter measurements to allow more optimal statistical analyses, but only 7 mock-treated animals were included in molecular analysis, since the treatment with the PPARα agonist would affect the gene expression levels. The PPARα adaptive capacity analysis will be covered in a separate publication. INT-exposed mice were sacrificed in their ad libitum MF feeding week.

After sedation with a mixture of isoflurane (1.5%), nitrous oxide (70%), and oxygen (30%), blood samples were collected by cardiac puncture, then followed by neck dislocation. The epidydimal white adipose tissue (WAT) and liver were weighed and were subsequently snap-frozen and stored at −80°C until further molecular and biochemical analyses. For histological analysis, the livers were fixed in 4% paraformaldehyde.

### 2.3. Oral glucose tolerance test

The mice sacrificed at the age of 12 months were all subjected to an oral glucose tolerance test (OGTT) 2 wk prior to sacrifice. In the OGTT, the mice were fasted for 6 h, then received 1.5 mg glucose per gram body weight via an oral gavage. Subsequently, blood glucose was measured 15, 30, 45, 60, 90, and 150 min following the glucose load using Accu-Check blood glucose meters (Roche Diagnostics, Almere, The Netherlands).

### 2.4. RNA isolation

Total RNA was isolated using TRIzol reagent (Invitrogen, Breda, The Netherlands) according to the manufacturer's instructions. The RNA was treated with DNAse and purified on columns using the RNeasy microkit (Qiagen, Venlo, The Netherlands). RNA concentration was measured on a NanoDrop ND-1000 UV–vis spectrophotometer (Isogen, Maarsen, The Netherlands) and RNA integrity was checked on an Agilent 2100 Bioanalyzer (Agilent Technologies, Amsterdam, The Netherlands) with 6000 Nano Chips according to the manufacturer's instructions. RNA was judged as suitable only if samples showed intact bands of 18S and 28S ribosomal RNA subunits, displayed no chromosomal peaks or RNA degradation products, and had a RNA integrity number (RIN) above 8.0.

### 2.5. Microarray hybridization and analysis

To reveal the liver transcriptomic profile of the four different diets, whole-genome gene expression was analyzed by microarray analysis. This analysis included seven mock-treated animals from each C, MF, and INT diet groups, and six animals from the CR diet group. One hundred nanograms of RNA was used for Whole Transcript cDNA synthesis (Affymetrix, Santa Clara, CA, USA). Hybridization, washing, and scanning of Affymetrix GeneChip Mouse Gene 1.1 ST arrays were carried out according to standard Affymetrix protocols. Arrays were normalized using the Robust Multiarray Average method [21,22]. Probe sets were defined according to Dai et al. [23]. In this method probes are assigned to unique gene identifiers, in this case Entrez IDs. The probes on the Gene 1.1 ST arrays represent 21225 Entrez IDs. For the analysis, only genes having intensity value of >20 on at least five array were taken into account, which resulted in 14 758 genes. Array data have been submitted to the Gene Expression Omnibus, with accession number GSE61233. The hierarchical clustering plot depicting gene expression profile similarity was constructed by using Multiple Experiment Viewer [24] and the accompanying body weight heatmap was prepared in Excel.

### 2.6. cDNA synthesis and real-time quantitative PCR

Real-time quantitative PCR (Q-PCR) was used to quantify gene expression changes for a selection of genes on all individual samples, as described previously [25]. For each individual sample, single-stranded complementary DNA (cDNA) was synthesized from 1 μg of total RNA using the first strand cDNA Synthesis Kit (Thermo Scientific, Landsmeer, The Netherlands) following the supplier's protocol. Primer sequences were retrieved from the online PrimerBank database [26], or otherwise designed using the Primer3 program [27] and the sequences of the primers used are listed in Supporting Information Table 2. Primers were tested for specificity by BLAST analysis. Q-PCR was performed using SensiMix SYBR No-ROX kit (Bioline, Alphen aan den Rijn, The Netherlands) and CFX384 thermal cycler (Bio-Rad, Veenendaal, The Netherlands). The following thermal cycling conditions were used: 2 min at 94°C, followed by 40 cycles of 94°C for 15 s, and 60°C for 45 s. Q-PCR reactions were performed in duplicate and all samples were normalized to 18S expression.

### 2.7. Histology

Paraffin-embedded liver tissues were cut at 5 μm thickness and mounted on Superfrost microscope slides. The sections were dewaxed in xylene and rehydrated in a series of graded alcohols. After staining with Meyer's haematoxylin-eosin (H-E) or fast green FCF/Sirius red F3B staining sections were mounted with DePex mounting medium (Gurr, Dorset, UK).

### 2.8. Hepatic TG content determination

Liver homogenates of 5% w/v were prepared in buffer containing 250 mM sucrose, 1 mM EDTA, 10 mM Tris-HCl (pH 7.5). Liver TG content was determined using the TG liquicolor monoreagent from Instruchemie (Delfzijl, The Netherlands) according to the manufacturer's instruction.

### 2.9. Plasma measurement

Plasma concentration of alanine aminotransferase (ALT) and aspartate aminotransferase (AST) were measured with commercially available kits from Instruchemie (Delfzijl, the Netherlands) following the protocol optimized by Stienstra et al. [28]. Plasma insulin level was measured using kit from ALPCO Diagnostics (Salem, NH, USA) according to the manufacturer's instruction.

### 2.10. Statistical analysis

Data analysis was performed with GraphPad Prism version 5.04 (GraphPad Software, San Diego, USA), using one-way ANOVA followed by Tukey post-test analysis. Correlation analysis was performed using Pearson correlation; *p* < 0.05 was considered significant.

## 3. Results

### 3.1. Modest body, liver, and WAT weight gain in mice exposed to an INT diet

**Figure 1:**
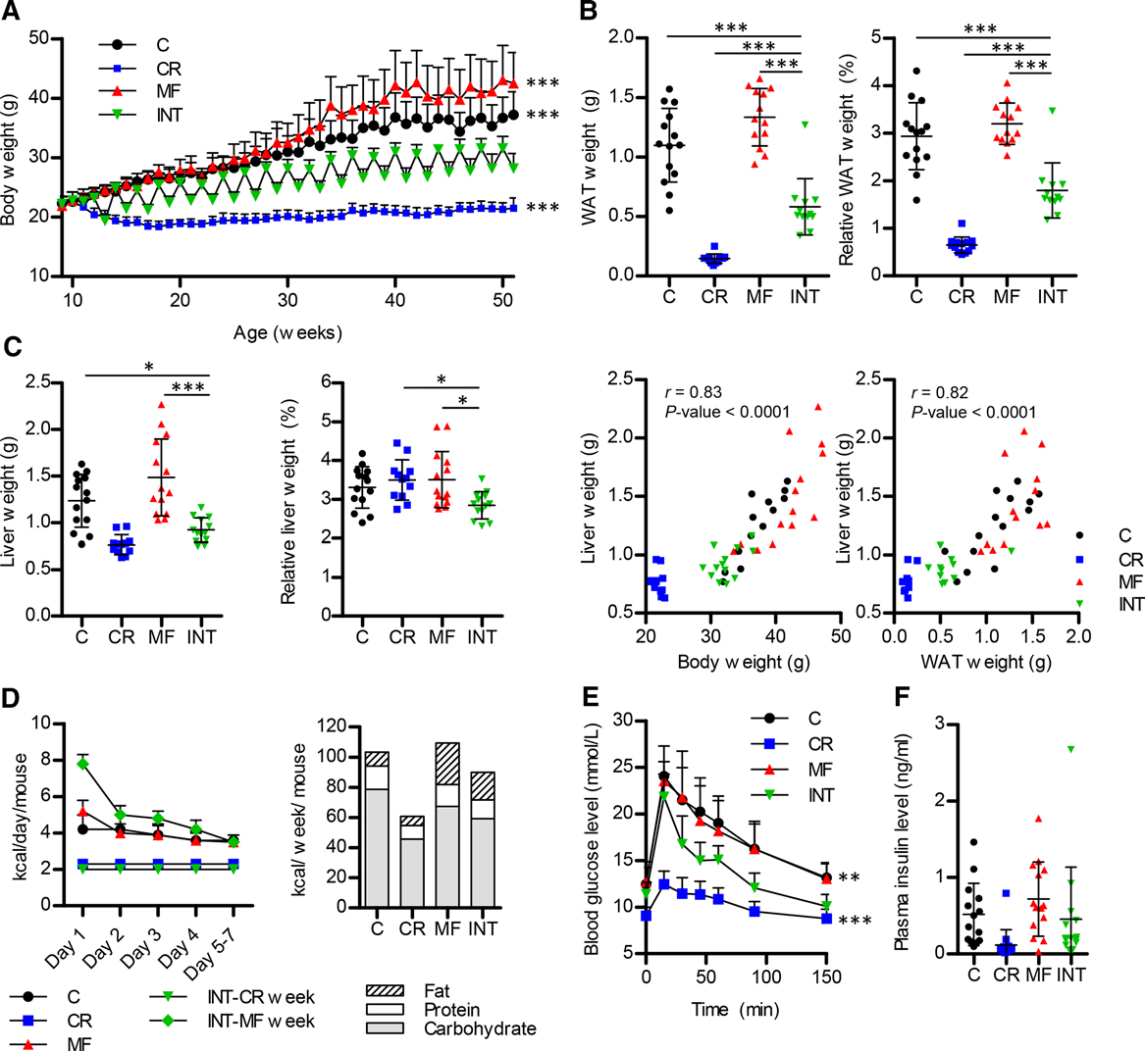
Beneficial effects of an INT diet regimen on body, WAT, and liver weight, food intake, and glucose tolerance. (A) Weekly measurement of body weight. (B) WAT weight and the ratio to the body weight. (C) Liver weight, ratio to body weight, and liver weight correlation with body and WAT weight. (D) Energy intake measurement at 12 months, daily (left) and weekly (right). (E) Glucose clearance measured by an oral glucose tolerance test. (F) Fasting plasma insulin levels. Error bars reflect standard deviation (SD). *p <0.05; **p < 0.01; ***p < 0.001 versus the INT-fed group.

To determine the physiological features of mice after exposure to the INT diet regimen, various parameters were measured. Adaptation to the high/low energy intake was observed in the weekly body weight measurements of the INT-fed mice, showing fluctuations in mean body weight related to the diet received in the preceding week (Fig. 1A). After the ad libitum feeding week body weight of the INT-fed mice was lower than that of the C and MF groups, while after the calorie restricted week their body weight remained higher than that of the mice in the CR group. Strong heterogeneity in body weight of mice in the C and MF diet groups was observed, which was reflected by the large error bars, in contrast to the mice within the CR and INT groups where the variation was very low.

Body weight gain in the C- and MF-fed mice included a significant increase in adiposity measured by the amount of white adipose tissue present in WAT, compared to the CR- and INT-fed mice (*p* < 0.001, Fig. 1B). Similarly, the WAT-to-body weight ratio also showed a significant increase in C and MF (*p* < 0.001). Liver weights of the INT-fed mice were nearly as low as that of the CR mice and differed significantly from the liver weights of the C and MF groups (*p* < 0.05 and <0.001, respectively, Fig. 1C). Interestingly, while the liver-to-body weight ratio of the C, CR, and MF groups were comparable, a slight but significant decrease was found for the INT-fed mice compared to CR- and MF-exposed mice (*p* < 0.05). Overall, a strong correlation between liver and body weight was observed (*r* = 0.83) and also between liver and WAT weight (*r* = 0.82).

### 3.2. Eating behavior and total energy intake in mice exposed to the INT diet

As anticipated, the switch between high/low food availability strongly affected the eating behavior of the INT-exposed mice. The daily energy intake measurement revealed hyperphagia during the first few days of the ad libitum feeding week (Fig. 1D), compensating the 40% energy reduction in the restricted feeding week, followed by a gradual decrease in food intake. As a result, the actual total energy intake of the INT-fed mice was 87.9% of the mean intake of the C-fed mice (Supporting Information Table 3A). Furthermore, since the INT-fed mice received the MF diet during the week they were fed ad libitum, overall these mice consumed more fat, 20.5% of the total energy intake, compared to 9% in the C group (Supporting Information Table 3B).

### 3.3. Tolerance to glucose was maintained in mice exposed to INT diet

To establish the effects of the different diets on glucose metabolism, an OGTT was carried out in the mice 2 wk prior to sacrifice. The maximal glucose level measured in the INT-exposed mice was lower and returned more rapidly to the basal fasting level than that of the C and MF groups (*p* < 0.01, Fig. 1E), though not to the extent as observed in the CR group. Similarly, the area under the curve (AUC) of the INT diet group was significantly lower than the C and MF groups (*p* < 0.001, Supporting Information Fig. 1), but not as low as the AUC of the CR diet group. Furthermore, averaged fasting plasma insulin level was lower in the INT-fed mice compared to mice that received the C and MF diets (Fig. 1F), although this effect did not reach statistical significance (*p* > 0.05) due to the large interindividual variation between the mice in the different intervention groups.

### 3.4. INT diet protected the liver from developing the biochemical and histological marks of NAFLD

**Figure 2:**
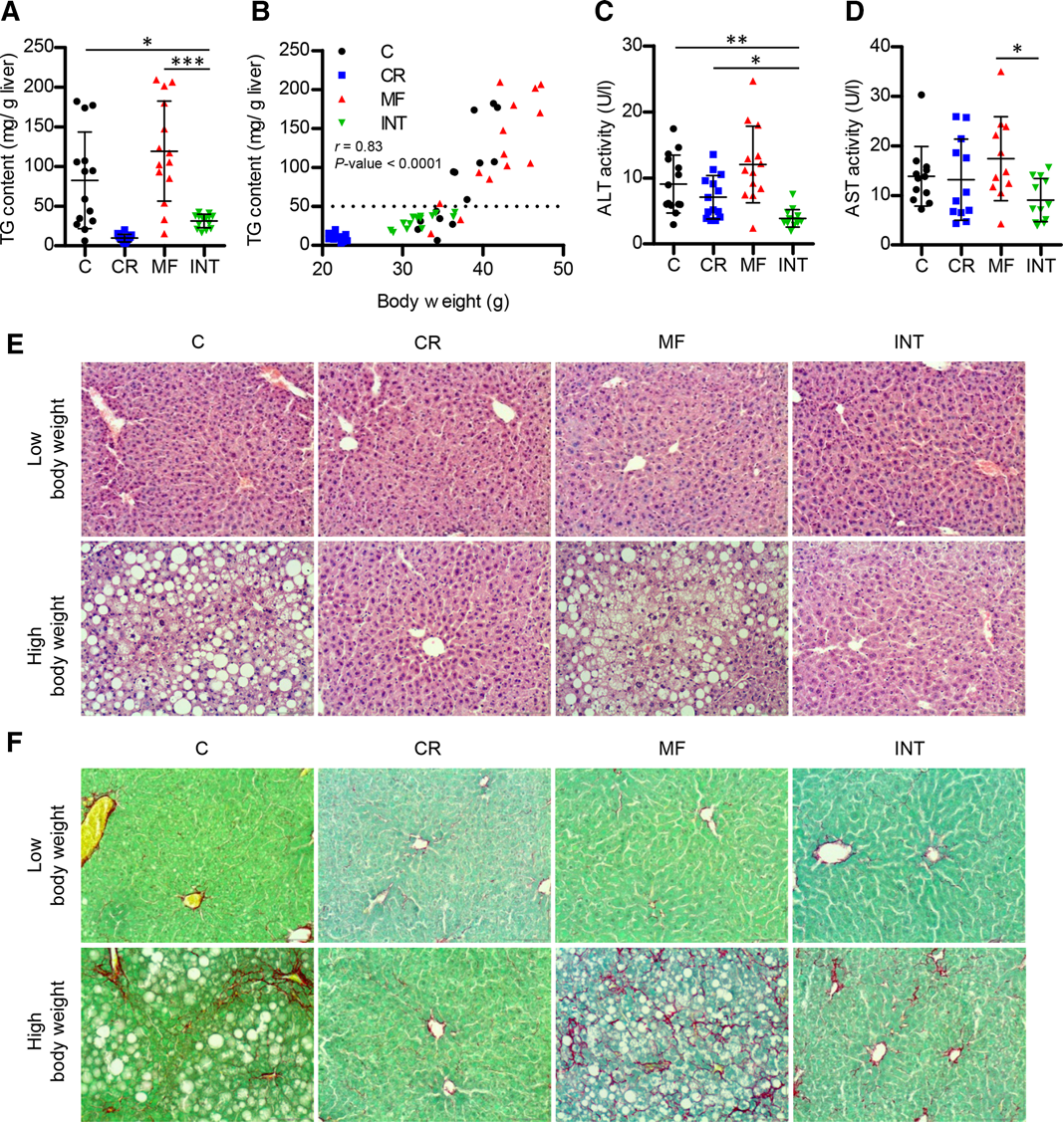
Hepatic TG, plasma ALT, and AST and liver histology indicated NAFLD development in C- and MF-fed mice, but not in mice exposed to the CR and INT diet. (A) Hepatic TG content. (B) Correlation between hepatic TG and body weight. (C) Plasma ALT. (D) Plasma AST. Error bars reflect SD. *p < 0.05; **p <0.01; ***p < 0.001 versus the INT group. (E) H–E staining and (F) FCF Green and Sirius Red staining of liver sections of animals with a high and low body weight (original magnification 200×).

Next, we evaluated the effects of the dietary interventions on various biochemical and morphological features related to the development of NAFLD. Fatty liver development was examined by quantification of the hepatic TG content. A very low TG content in mice exposed to the CR and INT diets was observed (Fig. 2A). Mice fed the C or MF diet showed hepatic TG deposition with a large interindividual difference, of which most of the animals exceed the diagnosis level of NAFLD, >5–10% (or 50–100 mg TG/ g liver) [29]. The level of TG content strongly correlated with body weight (*r* = 0.83, Fig. 2B) and liver weight (*r* = 0.91, Supporting Information Fig. 2A).

To determine whether hepatic function was affected due to exposure to the different diets, serum levels of ALT and AST, two well-established markers of liver injury/damage, were measured. Interestingly, the lowest ALT and AST levels were detected in the INT group (*p* < 0.05, Fig. 2C and D). In C- and MF-fed mice we found that the elevated level of the ALT was correlated with increasing hepatic TG levels (Supporting Information Fig. 2B).

Histological analysis was carried out to examine whether the different diets induced morphological changes in the liver. H-E staining of liver sections of C- and MF-fed mice with high body weights (the highest tertile of bodyweight), of which elevated hepatic TG levels were measured, revealed fat accumulation with development of macro- and micro-vesicular lipid droplets (Fig. 2E). On the other hand, animals with the low body weights (the lowest tertile of body weight) of the C and MF groups displayed normal liver morphology, similar to animals of the CR and INT intervention groups with both low and high body weights. To assess the progression of hepatic fibrosis, we performed a collagen Sirius-red staining (Fig. 2F). The results obtained showed that the high body weight-animals of the C and MF groups developed collagen scarring, indicating the presence of hepatic fibrosis in these mice, while this effect was not observed in CR and INT-fed mice.

### 3.5. Gene expression profiles reflected the body weight similarity amongst the C, MF, and INT groups, but not for the CR group

**Figure 3:**
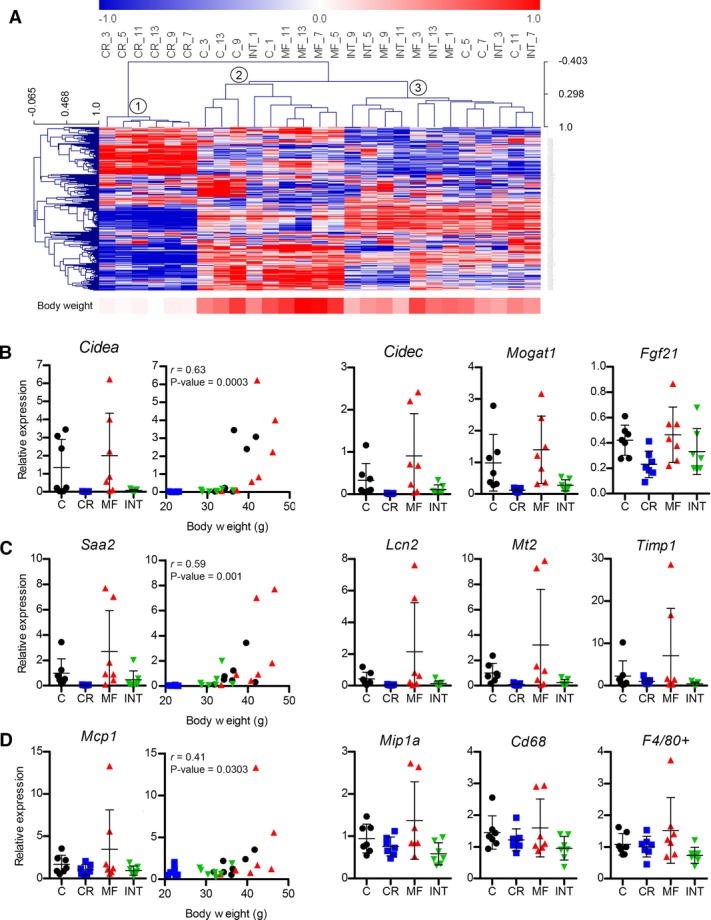
The INT-fed mice displayed a similar gene expression profile to the C- and MF-exposed mice with similar body weights. (A) A hierarchical clustering plot depicting the liver gene expression profiles similarity of the different diet regimens. The color band under the plot represents the body weight of individual mice in a white-to-red color scale (white = low values, red = high values). Q-PCR analysis on all animals of all dietary intervention groups shows differential expression of genes involved in (B) lipid droplet formation, (C) inflammatory and fibrosis, and (D) macrophage/monocyte recruitment. No statistical difference was found for gene expressions in the INT-fed mice compared to the other intervention groups. Error bars represent SD.

To obtain an overview of the molecular profiles induced by the different dietary interventions, a microarray analysis was carried out on the livers of the mice of all four intervention groups. An unsupervised clustering analysis was performed and a dendrogram plot of the gene expression profile similarity is depicted in Fig. 3A. The clustering analysis was performed on all genes, but due to space limitation the plot is represented by the top 250 most variable genes based on interquartile range. The dendrogram is complemented by a heatmap of the body weight, representing each animal. Three major clusters were observed: (1) a cluster of all animals in the CR diet group, (2) a cluster consisting of the C and MF animals that mostly contain a high body weight, and (3) a cluster that consists of animals with moderate body weight from the C, MF, and mostly INT diet group. The clustering and subclustering patterns of the second and third clusters suggested that the gene expression profile of the C, MF, and INT diet groups was strongly correlated with body weight similarity. Interestingly, the gene expression profile of the CR-exposed mice was distinct from those of the mice exposed to the INT diet, despite the fact that the physiological, biochemical, and morphological features of the mice in the CR and INT diet groups, e.g. hepatic TG content were similar.

### 3.6. Expression of genes associated with lipid droplet formation were increased with increasing body weight

To examine NAFLD development in the INT-fed animals at a molecular level in more detail, we analyzed the expression of genes involved in lipid droplet formation, which are known to be highly correlated with the severity of NAFLD [30–32]. Q-PCR analysis was performed on all mice of all four intervention groups for cell death-inducing DFFA-like effector A (*Cidea*) and C (*Cidec*), monoacylglycerol O-acyltransferase 1 (*Mogat1*), and fibroblast growth factor 21 (*Fgf21*; Fig. 3B). The expression levels of microarray and Q-PCR analysis corresponded to each other.

Mean expressions levels of the INT-fed mice for all four genes were lower than that of the C and MF groups. However, the observed differences did not reach statistical significance, most likely due to the large interindividual difference in expression levels observed in the C- and MF-fed mice, which was similar to the observed physiological features. Comparison of gene expression and body weight revealed significant positive correlations (*r* = 0.63–0.74) for all four genes (Fig. 3B and Supporting Information Fig. 3A). These results suggest that increased body weight might promote lipid droplet formation leading to NAFLD, while the INT regimen maintained a moderate body weight and low expression levels of genes involved in lipid droplet formation.

### 3.7. High body weight is essential but not sufficient for NASH pathogenesis

The effects of different diets on hepatic inflammation and fibrosis were assessed by Q-PCR analysis of four genes: serum amyloid A 2 (*Saa2*), lipocalin 2 (*Lcn2*), metallothionein (*Mt2*), and tissue inhibitor of metalloproteinase 1 (*Timp1*). The results presented in Fig. 3C revealed low expression levels of all four genes in all diet groups, except for a few animals in the C and MF groups that consistently showed elevated expression levels. Plotting of the gene expression levels versus body weight revealed that elevated expression levels of the inflammatory/fibrosis-related genes were exhibited by the C- and MF-exposed animals with the highest body weight (Fig. 3C and Supporting Information Fig. 3B), in which the positive correlations were considered significant (*p* < 0.0051–0.0102). However, not all animals with a high body weight showed elevated gene expression of the markers for inflammation and fibrosis, which was indicated by a weaker body weight-gene expression correlation compared with the lipid droplet formation genes (*r* = 0.48–0.59).

In addition, expression levels of previously established macrophage/monocyte marker genes linked to nonalcoholic steatohepatitis (NASH) pathogenesis [28], including monocyte chemoattractant protein 1 (*Mcp1*), macrophage inflammatory protein 1α (*Mip1α*), cell surface glycoprotein F4/80 (*F4/80^+^*), and CD68 antigen (*Cd68*), were analyzed. Expression levels of these macrophage/monocyte markers were generally low in all diet groups and an elevated level was only observed for a few animals in the MF group (Fig. 3D and Supporting Information Fig. 3C). It should be noted that elevated expression of inflammation and fibrosis genes was consistently found in the same animals having the highest body weight, explaining the weak correlations between the expression levels of monocyte/macrophage markers gene and body weight (*r* = 0.39–0.49).

Taken together, these results indicate that hepatic inflammation and macrophage/monocyte infiltration occur in only a few animals with high body weight mainly in the MF group. However, since not all mice having a high body weight showed these elevated gene expression levels of these markers, body weight is not necessarily an indicator of hepatic inflammation or infiltration.

## 4. Discussion

In this study, we showed that a weekly alternating diet consisting of 40E% CR and ad libitum feeding of a MF (25E%) diet prevented development of NAFLD/NASH, which was induced by life-long exposure to a MF diet and even the low-fat C diet. INT-fed mice maintained glucose tolerant, showed normal insulin levels, and low plasma ALT and AST levels. Furthermore, they did not exhibit signs of hepatic steatosis and fibrosis, indicated by the hepatic TG levels and morphology observations. Interestingly, all metabolic parameters measured were improved in the INT-fed mice and were found to be highly similar to the results obtained from mice that have been continuously exposed to a CR diet. Likewise, the INT and CR intervention groups exhibited no sign of hepatic lipid accumulation, inflammation, or macrophage/monocyte infiltration at the gene expression level.

Studies examining the effects of an increased dietary fat content on NAFLD development typically apply a high percentage of fat (40–60E%) to resemble the Western-style diet [33]. Hepatic steatosis is then acutely induced and developed at young age (3–4 months) [33]. However, the prevalence of NAFLD has been shown to increase with advancing age and this indicates that the adverse effect of Western diet accumulates over many years [34–37]. Therefore, in this study we applied a long-term exposure to a less extreme diet by using a fat content of 25E%, which we expected to simulate a slow onset of NAFLD developed at middle-age time point by the consumption of MF diet. We demonstrated that the long-term exposure of male C57BL/6J mice to a MF diet until the age of 12 months resulted in pronounced weight gain, impaired glucose clearance, elevated insulin levels, and an increase in plasma ALT levels. Hepatic steatosis and fibrosis were observed in some but not all mice which had the highest body weight. Together our results reveal that (i) long-term exposure to a MF diet seriously impairs metabolic homeostasis and is a risk factor for NAFLD development and (ii) applying every-other-week 40E% CR largely reversed the adverse health effects induced by the MF diet in 12-month-old mice.

Health-promoting effects of a CR diet have been commonly recognized, but compliance to such a diet is challenging for most individuals [15], and therefore an INT diet is explored as the alternative. Our results indicated that the adverse health effects of the MF diet could be reversed by applying every-other-week CR, however, it should be noticed that most metabolic parameters measured showed slightly better outcomes in the continuous CR-fed mice compared to the INT group. This result suggests that the INT regimen does not fully bestow all beneficial metabolic improvements of the CR diet. Only plasma ALT revealed significantly lower levels in INT-fed mice compared to mice that have received the CR diet. ALT is a transaminase enzyme and it has previously been shown that CR induces hepatic gluconeogenesis, accompanied by increased transamination activity as the first step in amino acid catabolism, resulting in elevated plasma ALT level [38]. In addition, it should be noted that although the outcomes of the INT diet regimen are novel as it discovers a remarkable improvement on liver parameters compared to an MF diet, we cannot exclude that the health benefits observed are (partially) due to lower body weights, in addition to the alternating of the diets.

We found that, despite the similarity in energy intake between the C and MF diet group, the animals that received a higher fat content in their diet developed a higher body weight. This has been observed in more studies [25,39] and possibly results from the lower energy requirement to store excess energy from fat than from carbohydrate, due to the additional conversion of glucose to fatty acids. However, our results demonstrate that body weight gain, and not the type of the diet, is a stronger predictor of hepatic steatosis development, e.g. liver mass, hepatic TG accumulation, and expression of genes associated with lipid droplet formation. Then, the development hepatic fibrosis and inflammation seem to be triggered by additional factor(s) in addition to body weight, as not all animals with the highest body weight developed fibrosis or inflammation, supporting the role of the “second hit” required in the development of NASH as first proposed by Day and James [40].

Substantial interindividual variation in all analyzed features was observed between mice of the C and MF groups. Heterogeneity in the response to diets is a previously reported characteristic of C57BL/6J mice [41,42]. We previously also showed that, upon exposure to a low- or high-fat diet, low or high responders could be distinguished in both intervention groups. The low and high responders revealed differences in food intake, body-weight gain, adiposity, a broad range of plasma markers, and liver features [30]. A number of possible mechanisms behind the observed variation in C57BL/6J mice have been proposed, such as copy number variations in the mouse genome and epigenetic features, but the exact underlying cause is not known yet [42,43].

**Figure 4:**
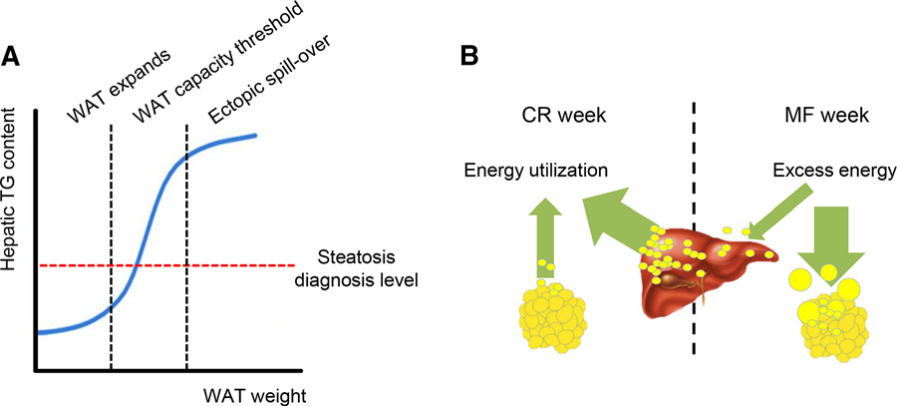
Proposed models for how the fat storage is distributed in WAT and liver. (A) Excess fat is initially stored in WAT, which may expand until a certain threshold. If the lipid-storage capacity is reached, ectopic fat deposition starts to occur in various organs, such as the liver. (B) During the ad libitum feeding week, the excess energy is mainly stored in WAT, but the compensation of deficit energy during the restricted feeding week might be mobilized predominantly from the liver fat storage. This proposed mechanism of an alternating diet may prevent hepatic steatosis development.

The results obtained in this study showed that in all four intervention groups both WAT and liver weight correlate, as expected, with body weight. However, the ratio of WAT- and liver-to-body weight in INT-exposed mice revealed an intriguing weight distribution. While relative WAT weight was higher than that of CR-fed mice, relative liver weight was significantly lower, suggesting a potential beneficial health effect for the liver of the INT compared to the CR diet. When more energy is consumed than needed for daily energy expenditure, the excess is in first instance stored in WAT, which has the capacity to expand in order to store the surplus of lipids and to prevent ectopic fat deposition. However, there is a limit to the lipid storage capacity of WAT and when this is exceeded, spillover is suggested to occur resulting in fat storage in organs, such as heart, pancreas, skeletal muscle, and liver [44]. When fat accumulation in the liver exceeds the threshold of 50–100 mg TG/ g liver it results in liver steatosis (Fig. 4A). Thus, the fact that the relative WAT weight was much higher than the relative liver weight in INT-fed mice suggests that energy excess consumed during the ad libitum MF week is primarily stored in the WAT, but the energy deficiency during the CR week is compensated by releasing fat from the liver, instead of fat utilization from WAT (as illustrated in Fig. 4B). This hypothesis is supported by a previous finding showing a rapid liver fat clearance (up to 29.6% decrease of liver TG content) occurring after 48 h of CR in humans [11]. Although the exact mechanism of the rapid liver fat extraction is unknown, this is an intriguing feature of the INT regimen that might contribute notably to NAFLD prevention by the INT diet.

Remarkably, our results revealed that while the metabolic profile of INT-fed mice was comparable to that induced by the CR diet, the INT- and CR-fed mice showed different transcriptomic profiles. As the INT-fed mice were sacrificed during their ad libitum MF feeding week, gene expressions patterns observed were most likely regulated to adapt to the ad libitum feeding condition to maintain homeostasis. This suggests that the transcriptome is a more flexible phenotype and more closely resembles the ad libitum-fed groups. Unfortunately, due to the limited number of animals in this study, we were not able to assess the liver transcriptomics of the INT-fed mice during their restricted feeding week.

In conclusion, the results obtained in this study show that a weekly alternating INT diet varying between 40E% CR and ad libitum MF feeding prevents NAFLD development in 12-month-old male C57BL/6J mice. Mice exposed to the INT diet retain healthy physiological features as displayed by continuous exposure to CR, while the gene expression profile was shown to be dissimilar with CR, but more similar to that of C- and MF-fed mice with comparable body weight.

## Abbreviations

ALT: alanine aminotransferase
AST: aspartate aminotransferase
C: control diet
CR: calorie restriction diet
H-E: haematoxylin-eosin
INT: intermittent
MF: medium fat
NAFLD: nonalcoholic fatty liver disease
NASH: nonalcoholic steatohepatitis
OGTT: oral glucose tolerance test
Q-PCR: real-time quantitative PCR
TG: triglyceride
WAT: epididymal white adipose tissue

## Supporting Information

As a service to our authors and readers, this journal provides supporting information supplied by the authors. Such materials are peer reviewed and may be re-organized for online delivery, but are not copy-edited or typeset. Technical support issues arising from supporting information (other than missing files) should be addressed to the authors.

Figure S1. The area under the curve (AUC) of oral glucose tolerance test (OGTT) of INT diet group significantly lower than the C and MF diet groups, but not to the extent of CR diet group.

**Figure S2A.** A correlation between liver TG content and liver weight Figure 2B. A correlation between plasma ALT and AST level to the liver TG content

**Figure S3A.** A correlation between body weight and expression of genes involved in lipid droplet formation **Figure S3B**. A correlation between body weight and expression of hepatic inflammation and fibrosis genes **Figure S3C**. A correlation between body weight and expression of macrophage/monocyte infiltration genes.

**Table S1. Composition of the experimental diet**. The CR diet was adjusted for the vitamins and minerals amount to ensure a homologous intake between both groups. **Table S2**. List of primer sequence used in Q-PCR analysis **Table S3A**. Relative energy intake of different diets **Table S3B**. Nutrient intake of the different diets
