# Supplementary material for: A weekly alternating diet between caloric restriction and medium fat protects the liver from fatty liver development in middle-aged C57BL/6J mice
Source: Mol Nutr Food Res. 2015 Jan 21;59(3):533–43. doi: 10.1002/mnfr.201400621 (PMC4681412; doi:10.1002/mnfr.201400621)
Supplement: Supplementary file 1 — Figure S1. The area under the curve (AUC) of oral glucose tolerance test (OGTT) of INT diet group significantly lower than the C and MF diet groups, but not to the extent of CR diet group. [file mnfr0059-0533-sd1.pptx]

## Slide 1
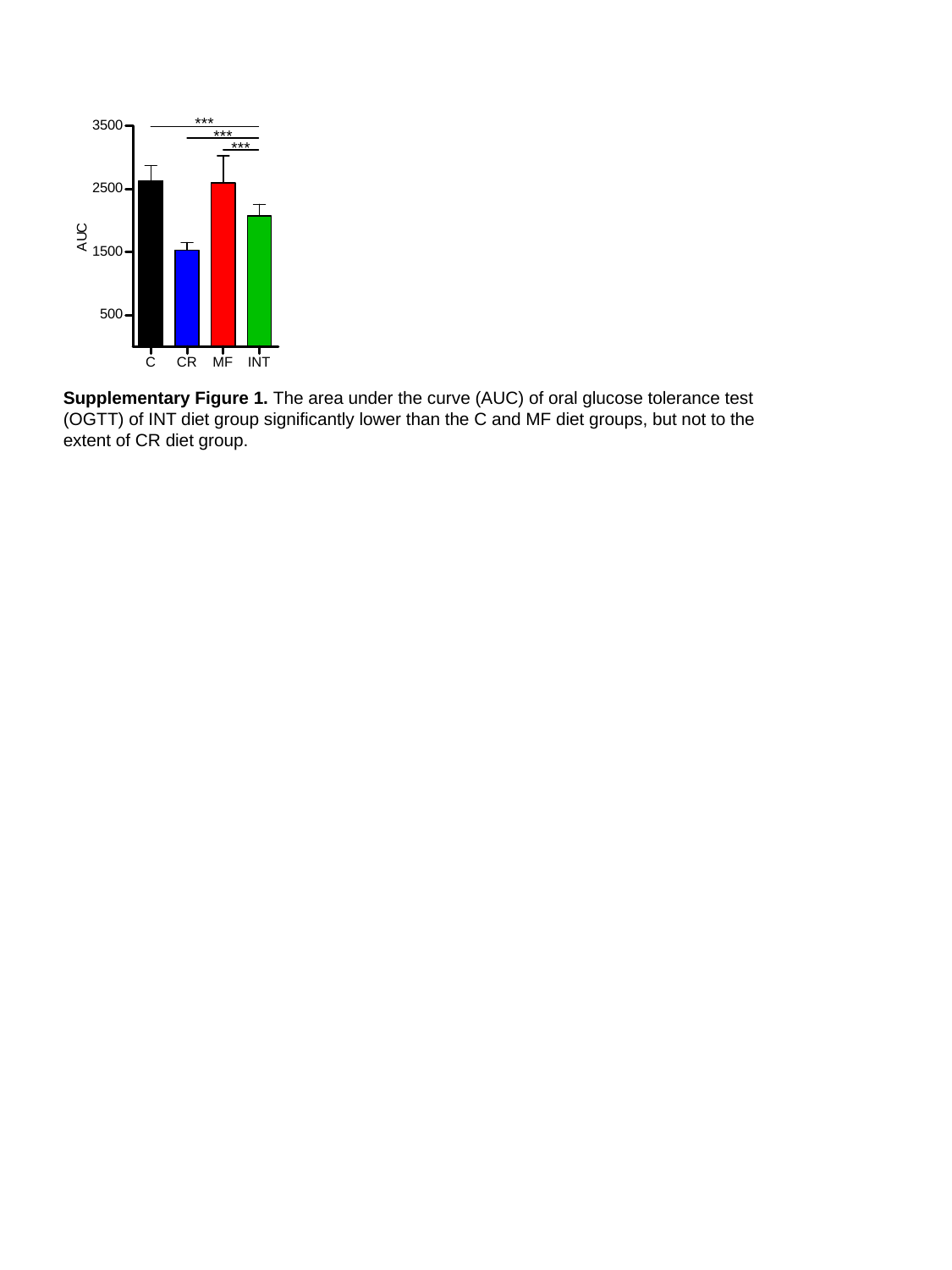

Supplementary Figure 1. The area under the curve (AUC) of oral glucose tolerance test (OGTT) of INT diet group significantly lower than the C and MF diet groups, but not to the extent of CR diet group.
