# Supplementary material for: A weekly alternating diet between caloric restriction and medium fat protects the liver from fatty liver development in middle-aged C57BL/6J mice
Source: Mol Nutr Food Res. 2015 Jan 21;59(3):533–43. doi: 10.1002/mnfr.201400621 (PMC4681412; doi:10.1002/mnfr.201400621)
Supplement: Supplementary file 2 [file mnfr0059-0533-sd2.pptx]

## Slide 1
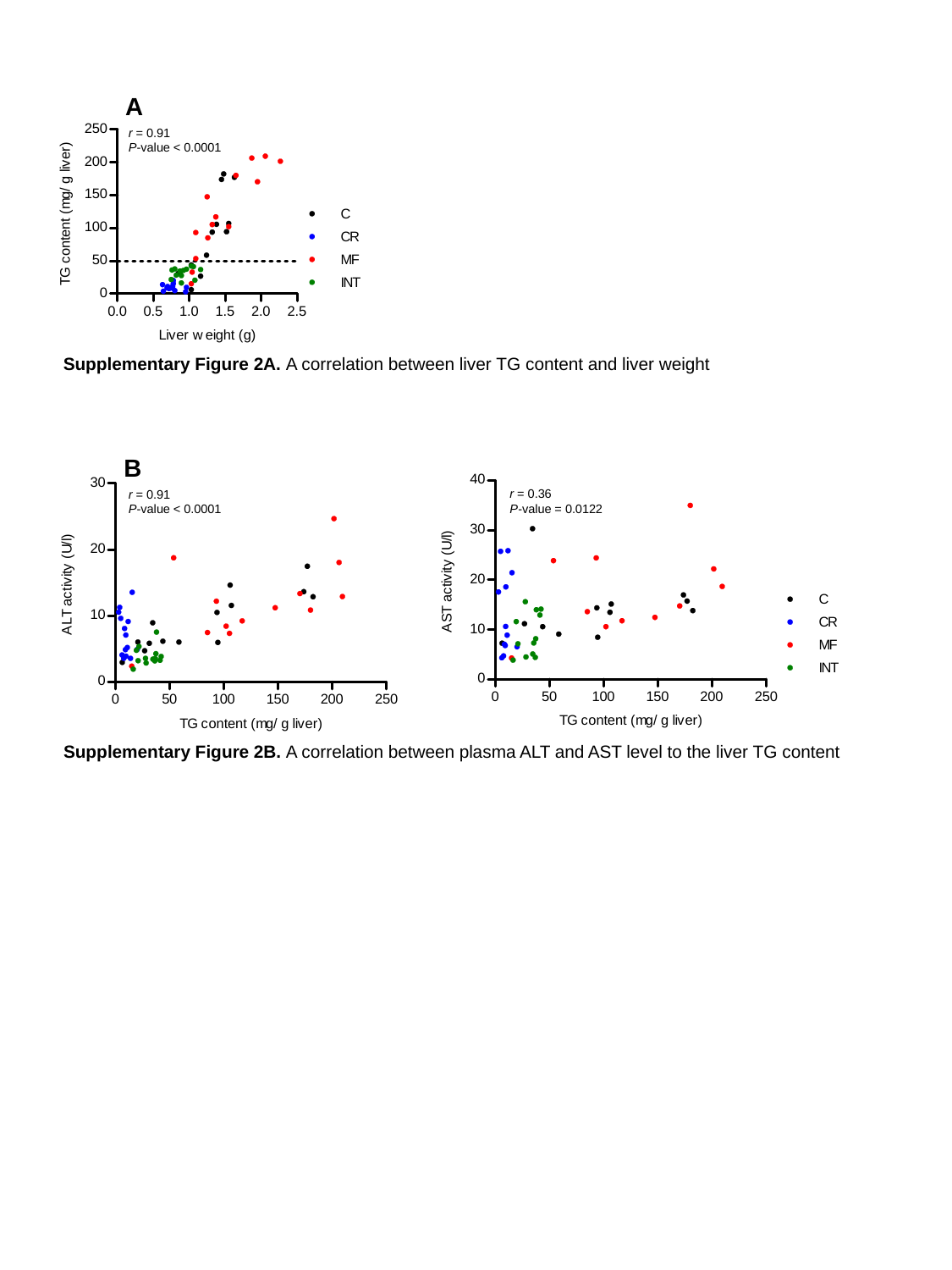

A
r = 0.91
P-value < 0.0001
Supplementary Figure 2A. A correlation between liver TG content and liver weight
B
r = 0.36
P-value = 0.0122
r = 0.91
P-value < 0.0001
Supplementary Figure 2B. A correlation between plasma ALT and AST level to the liver TG content
