# Supplementary material for: A weekly alternating diet between caloric restriction and medium fat protects the liver from fatty liver development in middle-aged C57BL/6J mice
Source: Mol Nutr Food Res. 2015 Jan 21;59(3):533–43. doi: 10.1002/mnfr.201400621 (PMC4681412; doi:10.1002/mnfr.201400621)
Supplement: Supplementary file 3 [file mnfr0059-0533-sd3.pptx]

## Slide 1
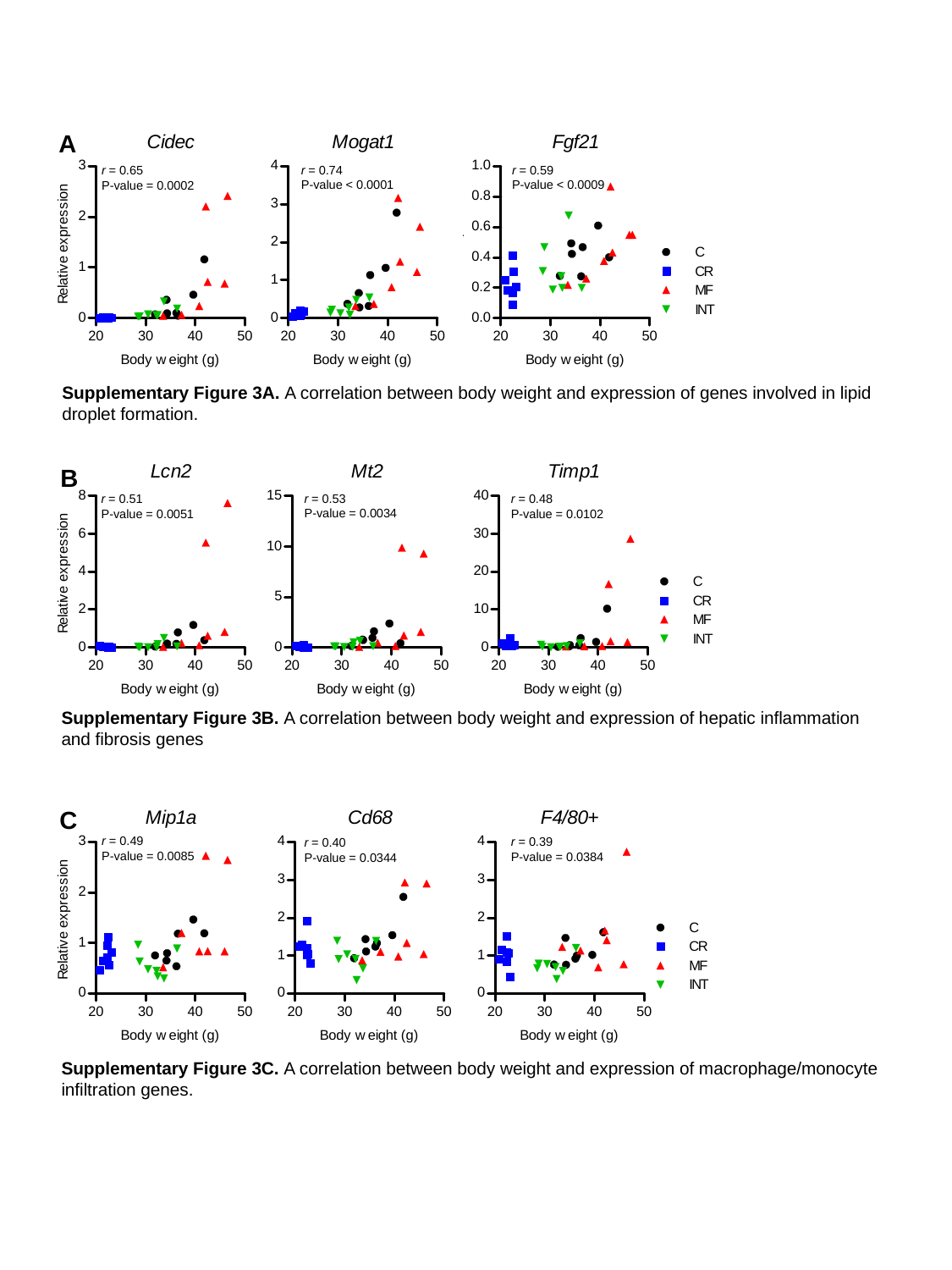

A
r = 0.74
P-value < 0.0001
r = 0.59
P-value < 0.0009
r = 0.65
P-value = 0.0002
Supplementary Figure 3A. A correlation between body weight and expression of genes involved in lipid droplet formation.
B
r = 0.53
P-value = 0.0034
r = 0.51
P-value = 0.0051
r = 0.48
P-value = 0.0102
Supplementary Figure 3B. A correlation between body weight and expression of hepatic inflammation and fibrosis genes
C
r = 0.49
P-value = 0.0085
r = 0.39
P-value = 0.0384
r = 0.40
P-value = 0.0344
Supplementary Figure 3C. A correlation between body weight and expression of macrophage/monocyte infiltration genes.
