# Supplementary material for: A weekly alternating diet between caloric restriction and medium fat protects the liver from fatty liver development in middle-aged C57BL/6J mice
Source: Mol Nutr Food Res. 2015 Jan 21;59(3):533–43. doi: 10.1002/mnfr.201400621 (PMC4681412; doi:10.1002/mnfr.201400621)
Supplement: Supplementary file 4 [file mnfr0059-0533-sd4.docx]

## SUPPLEMENTARY TABLES

**Supplementary Table 1. Composition of the experimental diet.** The CR diet was adjusted for the vitamins and minerals amount to ensure a homologous intake between both groups.

|  | **AIN-93W** | **AIN-93W-CR** | **AIN-93W-MF** |
| --- | --- | --- | --- |
| Energy (kcal/g) | 3.85 | 3.77 | 4.25 |
| Energy from fat (%) | 9 | 10 | 25 |
| Energy from protein (%) | 15 | 15 | 13 |
| Energy from carbohydrates (%) | 76 | 75 | 61 |
| Mineral mix AIN-93M (g%) | 35 | 50 | 35 |
| Vitamin mix AIN-93M (g%) | 10 | 14 | 10 |
| Choline bitartrate (g%) | 2.5 | 3.5 | 2.5 |

**Supplementary Table 2. List of primer sequence used in Q-PCR analysis**

| **Gene name** | **Forward primer (5’ → 3’)** | **Reverse primer (5’ → 3’)** |
| --- | --- | --- |
| *Cidea* | TGACATTCATGGGATTGCAGAC | GGCCAGTTGTGATGACTAAGAC |
| *Cidec* | ATGGACTACGCCATGAAGTCT | CGGTGCTAACACGACAGGG |
| *Mogat1* | TCCCGTTGTTCCGAGAATATCT | TGCTCAGCACATGAGACAAAC |
| *Fgf21* | GTGTCAAAGCCTCTAGGTTTCTT | GGTACACATTGTAACCGTCCTC |
| *Saa2* | GCGAGCCTACACTGACATGA | TTTTCTCAGCAGCCCAGACT |
| *Lcn2* | TGGAAGAACCAAGGAGCTGT | GGTGGGGACAGAGAAGATGA |
| *Mt2* | GCCTGCAAATGCAAACAATGC | AGCTGCACTTGTCGGAAGC |
| *Timp1* | GCAACTCGGACCTGGTCATAA | CGGCCCGTGATGAGAAACT |
| *Mcp1* | CCCAATGAGTAGGCTGGAGA | TCTGGACCCATTCCTTCTTG |
| *Mip1α* | CCTCTGTCACCTGCTCAACA | GTAGACTCACATGGCGCTGA |
| *F4/80^+^* | CTTTGGCTATGGGCTTCCAGTC | GCAAGGAGGACAGAGTTTATCGTG |
| *Cd68* | CCAATTCAGGGTGGAAGAAA | CTCGGGCTCTGATGTAGGTC |
| *18s* | CGGCTACCACATCCAAGGA | CCAATTACAGGGCCTCGAAA |

**Supplementary Table 3A. Relative energy intake of different diets**

|  | **Control** | **CR** | **MF** | **ID** |
| --- | --- | --- | --- | --- |
| Energy intake (kcal/week) | 103.2±9.4 | 60.8±1.1 | 116.9±12.0 | 90.7±8.1 |
| Relative energy intake (%) | 100 | 58.9 | 113.3 | 87.9 |

**Supplementary Table 3B. Nutrient intake of the different diets**

| **Nutrients** | | **Control** | **CR** | **MF** | **ID** |
| --- | --- | --- | --- | --- | --- |
| Carbohydrate | Kcal | 78.6±7.7 | 45.6±0.8 | 67.4±9.0 | 59.3±4.9 |
|  | % | 76.0 | 75.0 | 61.6 | 65.9 |
| Protein | kcal | 15.5±1.5 | 9.1±0.2 | 14.4±1.9 | 12.3±1.0 |
|  | % | 15.0 | 15.0 | 13.1 | 13.7 |
| Fat | Kcal | 9.3±0.9 | 6.1±0.1 | 27.6±3.7 | 18.4±2.0 |
|  | % | 9.0 | 10.0 | 25.3 | 20.5 |
